# Evaluation of Micro Satellite Instability and Mismatch Repair Status in Different Solid Tumors: A Multicenter Analysis in a Real World Setting

**DOI:** 10.3390/cells10081878

**Published:** 2021-07-24

**Authors:** Umberto Malapelle, Paola Parente, Francesco Pepe, Caterina De Luca, Pasquale Pisapia, Roberta Sgariglia, Mariantonia Nacchio, Gianluca Gragnano, Gianluca Russo, Floriana Conticelli, Claudio Bellevicine, Elena Vigliar, Antonino Iaccarino, Claudia Covelli, Mariangela Balistreri, Celeste Clemente, Giovanni Perrone, Angela Danza, Fabio Scaramuzzi, Matteo Fassan, Giancarlo Troncone, Paolo Graziano

**Affiliations:** 1 Department of Public Health, University of Naples Federico II, 80131 Naples, Italy; umberto.malapelle@unina.it (U.M.); francesco.pepe4@unina.it (F.P.); caterina.deluca@unina.it (C.D.L.); pasquale.pisapia@unina.it (P.P.); roberta.sgariglia@unina.it (R.S.); mariantonia.nacchio@unina.it (M.N.); gianluca.gragnano@unina.it (G.G.); gianlucar93@libero.it (G.R.); floriana.conticelli@unina.it (F.C.); claudio.bellevicine@unina.it (C.B.); elena.vigliar@unina.it (E.V.); antiaccc@hotmail.com (A.I.); 2 Unit of Pathology, Fondazione IRCCS Casa Sollievo Della Sofferenza, San Giovanni Rotondo, 71013 Foggia, Italy; paolaparente77@gmail.com (P.P.); cla.covelli85@gmail.com (C.C.); celesteclemente@alice.it (C.C.); pergianni63@libero.it (G.P.); angelamariadanza1962@gmail.com (A.D.); fabio3164@hotmail.it (F.S.); p.graziano@operapadrepio.it (P.G.); 3 Surgical Pathology Unit, Department of Medicine (DIMED), University of Padua, 35128 Padua, Italy; mariangela.balistreri@aopd.veneto.it (M.B.); matteo.fassan@unipd.it (M.F.); 4 Veneto Institute of Oncology, IOV-IRCCS, 35128 Padua, Italy

**Keywords:** predictive molecular pathology, IHC, fully automated RT-PCR, microfluidic, MMR, MSI, immunotherapy, immune checkpoint inhibitors (ICIs)

## Abstract

Immune-checkpoint inhibitors (ICIs) play a key role in the treatment of advanced stage colorectal cancer (CRC) patients featuring a deficient DNA mismatch repair (dMMR) system or a high microsatellite instability (MSI-H) profile. However, beyond the established role in CRC patients, ICIs have highly proven efficacy in other solid tumors featuring MSI-H/dMMR status represented by endometrial, gastric, ovarian, prostatic, and pancreatic carcinomas (EC, GC, OC, PrC, and PaC). Our aim was to compare the concordance rates among the Idylla™ MSI test, TapeStation 4200, and immunohistochemical (IHC) analysis in assessing MSI-H/dMMR status in EC, GC, OC, PrC, and PaC patients. The Sanger sequencing-based Titano MSI test was used in discordant cases. One hundred and eighty-five cases (*n* = 40 PrC, *n* = 39 GC, *n* = 38 OC, *n* = 35 PaC, and *n* = 33 EC) were retrospectively selected. MMR protein expression was evaluated by IHC. After DNA quality and quantity evaluations, the Idylla^TM^ and TapeStation 4200 platforms were adopted for the evaluation of MSI status. Remarkably, compared to IHC, the Idylla™ platform achieved a global concordance rate of 94.5% (154/163) for the microsatellite stable (MSS)/proficient MMR (pMMR) cases and 77.3% (17/22) for the MSI-H/dMMR cases. Similarly, a global concordance rate of 91.4% (149/163) and 68.2% (15/22) for MSS/pMMR and MSI-H/dMMR cases was also identified between IHC and the TapeStation 4200 microfluidic system. In addition, a global concordance of 93.1% (148/159) and 69.2% (18/26) for MSS/pMMR and MSI-H/dMMR cases was observed between the Idylla™ and TapeStation 4200 platforms. Discordant cases were analyzed using the Titano MSI kit. Overall, our data pinpointed a central role for molecular techniques in the diagnostic evaluation of dMMR/MSI-H status not only in CRC patients but also in other types of solid tumors.

## 1. Introduction

DNA mismatch repair (MMR) is a highly conserved system responsible for restoring mismatching errors, such as single base mismatches, or small insertions and deletions. When these errors are not corrected, owing to a flawed MMR system, genomic steadiness is disrupted during DNA replication and recombination—a phenomenon that eventually gives rise to multiple cancer-associated mutations [1,2,3,4,5]. Generally, a deficient MMR (dMMR) system, is triggered by germline (e.g., Lynch syndrome), somatic, and epigenetic changes, which, in turn, result in the inactivation of MMR genes [1,3,6,7,8,9]. Thus, identifying MMR defective genes is crucial for cancer patient management.

The status of the MMR system is generally assessed by immunohistochemical analysis of the four proteins involved in MMR: namely, MLH1, MSH2, MSH6 and PMS2 [1,10]. However, evaluation and interpretation of immunohistochemical results may at times be challenging because of intra- and inter-observer variability and pre-analytical and analytical issues [1]. In this scenario, evaluation of microsatellite instability (MSI) through molecular approaches, such as polymerase chain reaction (PCR), genomic sequencing, and capillary electrophoresis, has been proposed as a valuable alternative to overcome some of the issues inherent to immunohistochemistry (IHC) [1,11].

In brief, during DNA replication, microsatellites, or short tandem mono or dinucleotide repeated sequences, which are widely disseminated in both coding and non-coding regions of the genome, may be susceptible to errors that are usually corrected by the MMR complex [12]. Generally, the most widely adopted procedure for MSI evaluation relies on the Sanger sequencing-based Bethesda panel, which covers two mononucleotide (*BAT-25* and *BAT-26*) and three dinucleotide (*D5S346*, *D2S123,* and *D17S250*) repetitions [13] or, alternatively, a panel covering five poly-A mononucleotide repeats (*BAT-25*, *BAT-26*, *NR-21*, *NR-24*, *NR-27*) [14]. However, recent studies have demonstrated that the fully automated PCR high-resolution melt curve analysis (Idylla^TM^, Biocartis, Mechelen, Belgium) [11,15,16,17,18,19,20,21,22,23,24,25,26] and the automated microfluidic electrophoretic run chip-based assay (TapeStation 4200, Agilent Technologies, Santa Clara, CA, USA) are easier to use, faster, and less expensive than Sanger sequencing [11,27,28]. 

Clinically, MMR/MSI testing has attracted increasing interest following Food and Drug Administration (FDA) approval in 2017 of the anti-programmed cell death protein 1 (PD-1) immune checkpoint monoclonal antibody pembrolizumab for MSI high (MSI-H)/dMMR patients with unresectable or metastatic solid tumors, regardless of age and histotype [29,30]. 

Beyond the established role of immune checkpoint inhibitors (ICIs) in the management of colorectal cancer (CRC) patients, these anticancer drugs have proven highly effective in other solid tumors featuring MSI-H/dMMR status. Among these, endometrial cancer (EC) displays a higher incidence of MSI-H status in endometrioid histotypes (40%) than in serous ones (2%) [31]. Overall, about 30% of primary ECs, and 13% to 30% of recurrent ECs, are MSI-H/dMMR [32]. Regarding the response rates to ICIs, MSI-H/dMMR advanced EC patients show higher response rates (from 27% to 57%) compared to microsatellite stable (MSS) patients (from 3% to 23%) [32]. 

A positive response to ICIs has also been reported in gastric carcinoma (GC) patients, who in 20% of cases feature MSI-H [33]. Indeed, MSI-H GC patients show a higher objective response rate (ORR) and disease control rate (DCR) to ICIs than MSS GC patients [34]. On the other hand, a lower frequency (12%) of MSI-H/dMMR status has been seen in unselected ovarian cancers (OCs) [35,36,37]. Regarding treatment response, more studies are needed to validate the efficacy of ICIs in MSI-H/dMMR OCs, suggesting that for this type of cancer, a better treatment option would be a combination therapy of ICIs and other anticancer drugs [38,39]. In intraductal papillary mucinous neoplasm (IPMN) of the pancreas, the incidence of MSI-H/dMMR is lower, dropping to 6.9% [40]. The lowest incidence rates of MSI-H/dMMR status have, instead, been observed in prostatic (PrC, about 3%) and pancreatic (PaC, about 1%) cancers [41,42]. However, despite such low incidence, ICIs have proven highly effective in both PrC and PaC patients featuring an MSI-H/dMMR status [41,42].

Thus, this evidence strongly underlines the importance of expanding the evaluation of MSI-H/dMMR status to different types of solid tumors. Although our research group has demonstrated the feasibility of adopting the fully automated PCR high-resolution melt curve analysis (Idylla^TM^) and the automated microfluidic electrophoretic run chip-based assay (TapeStation 4200) to assess MSI-H/dMMR status in CRC patients [11], little is yet known about their performance in other types of tumors. In an attempt to fill this knowledge gap, we compared the concordance rates between these two molecular approaches and IHC analysis in assessing MSI-H/dMMR status in EC, GC, OC, PrC, and PaC patients. The Sanger sequencing-based Titano MSI test (Diatech Pharmacogenetics, Jesi, Italy) was used to confirm discordant cases.

## 2. Materials and Methods

### 2.1. Study Design

For our comparative analysis, we retrospectively selected a total of *n =* 199 formalin fixed paraffin embedded (FFPE) (*n* = 40 PrC, *n* = 40 GC, *n* = 39 OC, *n* = 40 PaC, and *n* = 40 EC) tumor samples, analyzed by IHC for MMR protein expression at the Fondazione IRCCS Casa Sollievo della Sofferenza (San Giovanni Rotondo, Italy) from 2015 to 2020. The percentage of neoplastic cells and the pathological status of each patient were evaluated by two expert pathologists. Briefly, for the molecular evaluation of MSI, tumor and a corresponding normal tissue specimen were collected after revision of original hematoxylin-and-eosin (H&E)-stained sections. All information regarding human material was managed using anonymous numerical codes, and all samples were handled in compliance with the Declaration of Helsinki (http://www.wma.net/en/30publications/10policies/b3/ last accessed 25 May 2021).

### 2.2. Immunohistochemical Analysis

Expression of MMR proteins (MLH1, PMS2, MSH2, and MSH6) was evaluated by IHC, as previously reported [11].

Briefly, 3-µm thick FFPE tissue sections were deparaffinized in xylene, rehydrated in graded alcohols, washed in double-distilled water, and pretreated with DAKO solution (EnVision FLEX Target Retrieval Solution, High pH 50×) at 97 °C. The slides were then incubated with primary monoclonal antibodies against MLH1 (clone ES05 diluted 1:50, DAKO), PMS2 (clone EP51 diluted 1:40, DAKO), MSH2 (clone FE11 diluted 1:50, DAKO), and MSH6 (clone EP49 diluted 1:50, DAKO) for 30 min. The analysis was performed on the automated platform Autostainer Link 48 (Dako, Carpinteria, CA, USA) according to the manufacturer’s instructions. The antigen-antibody reaction was inspected with the EnVision FLEX kit with diaminobenzidine as chromogen. 

**Figure 1:**
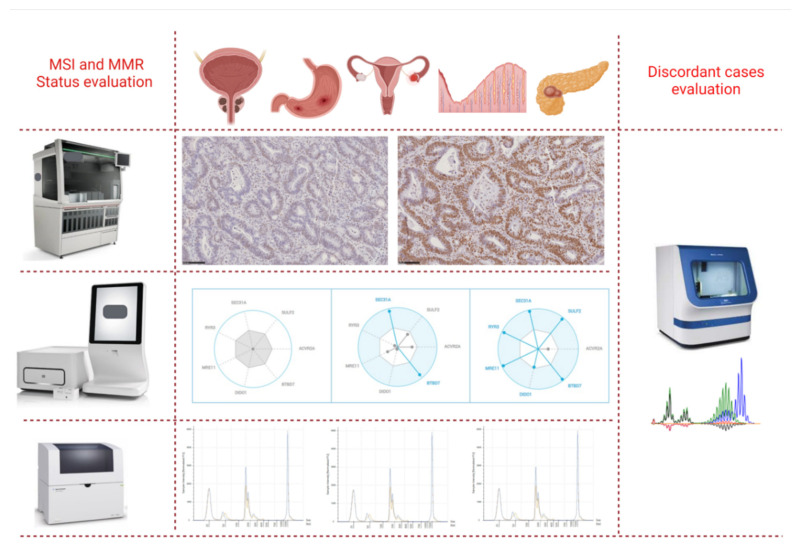
We retrospectively selected a total of *n* = 185 formalin fixed paraffin embedded (*n* = 40 prostate, *n* = 39 gastric, *n* = 38 ovarian, *n* = 35 pancreatic, and *n* = 33 endometrial) tumor samples, previously analyzed by immunohistochemistry for mismatch repair protein expression. In all samples, microsatellite instability (MSI) status was evaluated by Idylla™ MSI test and TapeStation 4200. Discordant cases were further analyzed by Titano MSI test analysis.

MMR protein expression was categorized as (i) retained (i.e., proficient MMR; pMMR), when a moderate to strong nuclear protein expression was detected in tumor cells as well as in internal control; and (ii) lost (i.e., dMMR), when a complete loss of nuclear expression in tumor cells was observed, but retained in normal cells [43]. Tumor samples showing absence of immunoreactions in internal controls were classified as “inadequate” for IHC evaluation and analysis and excluded from the study. A total of 185 cases were submitted to molecular approach (Figure 1).

### 2.3. DNA Extraction and Qualification

DNA extraction and qualification were performed as previously reported [11]. 

Overall, four 5-µm thick sections obtained from tumor tissues and corresponding normal mucosae were adopted. The neoplastic cell area was manually microdissected. Then, DNA was extracted with the Mini Amp kit (Qiagen, Hilden, Germany) following the manufacturer’s instructions. Finally, it was eluted in 30 µL of DNAse- and RNAse-free water (Thermo Fisher Scientifics, Waltham, MA, USA) and stored at −20 °C until DNA was qualified by the TapeStation 4200 microfluidic platform. In particular, the genomic ladder and sample buffer (Agilent Genomic ScreenTape, Agilent Technologies) were performed on the Genomic Screen Tape device (Agilent Technologies) according to the manufacturer’s instructions (Figure 1).

### 2.4. Microfluidic Analysis for MSI Status Evaluation

TapeStation 4200 analysis was performed as previously reported [11]. 

Five amplification reaction mixtures were prepared to analyze patients’ MSI status by using the Bethesda panel, starting from 20 ng of extracted DNA on the TapeStation 4200 system, as previously validated [11]. Briefly, for each patient, 1 µL of each amplified product from both tumor and normal tissues of each reaction mixture and 3 µL of D1000 Buffer (Agilent Technologies) were automatically charged on a solid device comprising 16 nanocapillaries (D1000 ScreenTape) and analyzed on the TapeStation 4200 platform. Results were inspected with proprietary software (TapeStation Analysis Software, Agilent Technologies) (Figure 1).

### 2.5. Idylla™ MSI Assay

The Idylla™ MSI test was performed as previously reported [11]. 

This test consists of a fully automated RT-qPCR system able to detect microsatellite instability directly from FFPE human cancer tissue sections. Overall, a total of four 5-micron thick slides obtained from tumor specimens were used for MSI analysis, according to the manufacturer’s instructions. Briefly, tumor areas were scraped from each sample with a sterile blade and inserted into an MSI cartridge, as previously described [11]. Overall, DNA was automatically extracted by a combination of HIFU, enzymatic/chemical digestion and heat, and then amplified into 5 PCR chambers. The Idylla MSI assay then analyzed homopolymers in 7 biomarkers (*ACVR2A*, *BTBD7*, *DIDO1*, *MRE11*, *RYR3*, *SEC31A*, and *SULF2*). Fluorescent signals, generated by fluorescently labeled molecular beacon probes, were automatically inspected by proprietary software able to analyze a minimum allele frequency of 10% and to calculate a probability score (MSI score) for all the tested biomarkers. Overall, samples with ≥2 of the 7 mutated biomarkers and <2 of the 7 mutated biomarkers were classified as MSI-H and MSS, respectively (Figure 1).

### 2.6. Discordant Cases Evaluted with the Titano MSI Test

The Titano MSI test analysis was performed as previously reported [11]. Briefly, the Titano MSI test (Diatech Pharmacogenetics) was used to investigate discordant cases between IHC and the 2 molecular approaches adopted in the study. DNA obtained from tumor and corresponding normal mucosa samples for each case were analyzed with MSI Titano kit. In brief, this test detects MSI status in cancer tissues through multiplex amplifications with fluorescent primers and subsequent DNA fragment analysis on an automated sequencer. An input of optimal 20 ng of extracted DNA is generally required to analyze variations in the number of microsatellite loci of 10 different molecular targets (*BAT25*, *BAT26*, *D2S123*, *D17S250*, *D5S346*, *BAT40*, *D18S58*, *NR21*, *NR24*, *TGFβRII*). In essence, variations are identified by comparing the peak profiles generated by capillary electrophoresis runs of tumor and corresponding normal tissue samples from each patient (Figure 1). 

## 3. Results

Overall, IHC analysis was successfully carried out in *n* = 185/199 (93.0%) formalin fixed paraffin embedded (FFPE) tumor samples (*n* = 40/40 PrC, *n* = 39/40 GC, *n* = 38/39 OC, *n* = 35/40 PaC, and *n* = 33/40 EC). DNA qualification revealed a median DNA concentration of 70.9 (ranging from 2.0 to 424.0 ng/µL) for the tumor samples and a median DNA concentration of 40.4 ng/µL (ranging from 0.1 to 60.0 ng/µL) for the normal samples. In detail, a median of 137.0 ng/µL (ranging from 13.2 to 414.0 ng/µL) and 38.2 ng/µL (ranging from 2.3 to 60.0 ng/µL), 90.8 ng/µL (ranging from 6.6 to 343.0 ng/µL) and 49.2 ng/µL (ranging from 9.5 to 60.0 ng/µL), 32.7 ng/µL (ranging from 2.1 to 179.0 ng/µL) and 31.8 ng/µL (ranging from 3.1 to 60.0 ng/µL), 31.0 ng/µL (ranging from 2.4 to 182.0 ng/µL) and 49.3 ng/µL (ranging from 6.3 to 60.0 ng/µL), 64.5 ng/µL (ranging from 4.4 to 187.0 ng/µL) and 35.7 ng/µL (ranging from 0.1 to 60.0 ng/µL) was evaluated for OC, EC, PrC, PaC, and GC tumor and normal mucosa specimens, respectively. In addition, a DNA integrity number (DIN) median value of 3.6 (ranging from 1.2 to 5.9) was globally observed in 183/185 (98.9%) cases. In particular, our analyses revealed a DIN median value of 4.3 (ranging from 1.9 to 5.9) for OC, of 3.4 (ranging from 1.9 to 5.6) for EC, of 3.8 (ranging from 2.3 to 5.3) for PrC of 2.8 (ranging from 1.2 to 4.8) for PaC, and of 3.7 (ranging from 2.4 to 5.3) for GC patients. IHC analysis highlighted an overall proficient MMR (pMMR) and dMMR status in 163/185 (88.1%) and 22/185 (11.9%) cases, respectively. In detail, 36/38 (94.7%) and 2/38 (5.3%), 27/33 (81.8%) and 6/33 (18.2%), 37/40 (92.5%) and 3/40 (7.5%), and 28/39 (71.8%) and 11/39 (28.9%) pMMR and dMMR cases were identified in OC, EC, PrC, and GC patients, respectively. All cases showed a pMMR profile in PaC samples. 

**Figure 2:**
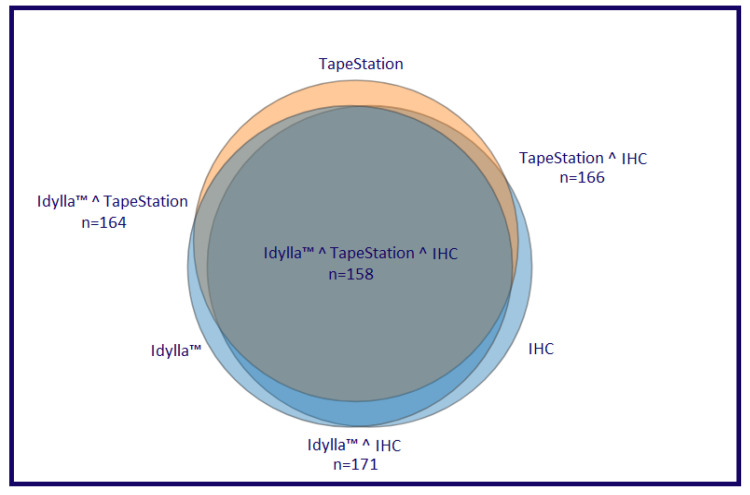
Venn diagram representing the concordance rate among the technologies adopted in the present study. Briefly, dark gray circle represents fully concordant cases among the three different assays; dark gray plus dark orange circles represent the concordant cases between TapeStation 4200 and IHC; dark gray and dark blue circles represent the concordant cases between Idylla^TM^ and IHC.

**Figure 3:**
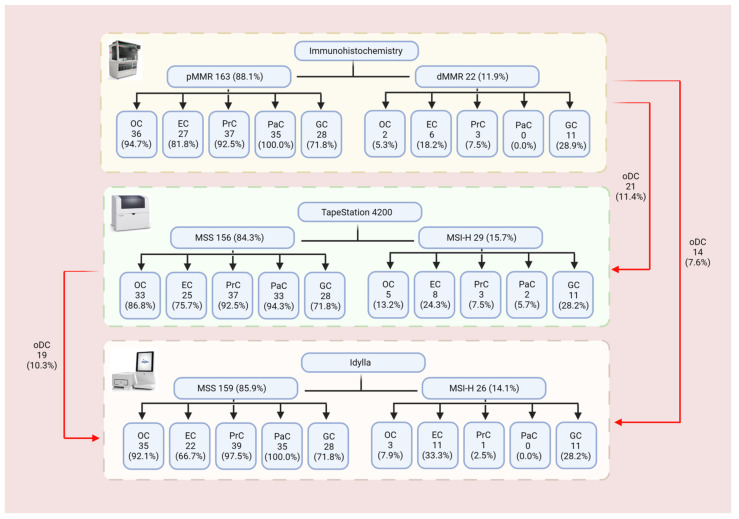
Flow chart summarizing results obtained with the three different assays. Abbreviations: dMMR: deficient mismatch repair; EC: endometrial cancer; GC: gastric cancer; MSI-H: high microsatellite instability; MSS: microsatellite stable; OC: ovarian cancer; oDC: overall discordant cases; PaC: pancreatic cancer; pMMR: proficient mismatch repair; PrC: prostatic cancer.

Similarly, the Idylla™ platform and TapeStation 4200 system globally detected an overall MSS profile in 159/185 (85.9%) and 156/185 (84.3%) cases, respectively, whereas a global MSI-H profile was identified in 26/185 (14.1%) and in 29/185 (15.7%) cases, respectively. Regarding, the MSS status detected by the TapeStation 4200 system, 35/156 (22.4%) cases displayed a low MSI (MSI-L) status. Moreover, an MSI-H profile was respectively detected in 3/38 (7.9%) and in 5/38 (13.2%) of OC patients; in 11/33 (33.3%) and in 8/33 (24.3%) of EC patients; in 1/40 (2.5%) and 3/40 (7.5%) of PrC patients; in 11/39 (28.2%) and in 11/39 (28.2%) of GC patients by using the Idylla™ and TapeStation 4200 platforms, respectively. The remaining cases showed an MSS profile. Among PaC cases, Idylla™ detected an MSS profile in all cases, whereas TapeStation 4200 detected 33/35 (94.3%) MSS cases and 2/35 (5.7%) MSI-H cases. Regarding the MSS cases, TapeStation 4200 detected 9/38 (23.7%), 2/33 (6.1%), 13/40 (32.5%), 6/39 (15.4%), and 5/35 (14.3%) MSI-L cases in OC, EC, PrC, GC, and PaC, respectively. Remarkably, compared to IHC, the Idylla™ platform achieved a global concordance rate of 94.5% (154/163) for the MSS/pMMR cases and 77.3% (17/22) for the MSI-H/dMMR cases. Similarly, a global concordance rate of 91.4% (149/163) and 68.2% (15/22) for MSS/pMMR and MSI-H/dMMR cases was also identified between IHC and the TapeStation 4200 microfluidic system. In addition, a global concordance of 93.1% (148/159) and 69.2% (18/26) for MSS/pMMR and MSI-H/dMMR cases was also observed between the Idylla™ and TapeStation 4200 platforms. Concordant results between the five histological groups are reported in Table 1, Table 2, Table 3, Table 4 and Table 5 and Figure 2 and Figure 3. 

Discordant cases among the TapeStation 4200 system, Idylla^TM^ platform, and IHC approach were successfully analyzed by applying the Titano MSI kit. The results showed that in 14 out of 27 (51.8%) cases, the Titano kit and IHC showed concordant results (*n* = 10 MSS/pMMR and *n* = 4 MSI-H/dMMR); whereas in 13 out 27 cases (48.2%), a discordant result was reported (*n* = 7 MSS/dMMR and *n* = 6 MSI-H/pMMR). As far as the comparison between the Titano MSI kit and Idylla^TM^ is concerned, overall, 22 concordant cases (81.5%; *n* = 13 MSS and *n* = 9 MSI-H) were detected. Only five discordant cases (*n* = 4 MSS for Titano MSI kit and MSI-H for Idylla^TM^ and *n* = 1 MSI-H for Titano MSI kit and MSS for Idylla^TM^) were reported. The results showed that in 11 out of 27 (40.7%) cases, the Titano MSI kit and TapeStation 4200 showed concordant results (*n* = 6 MSS and *n* = 5 MSI-H); whereas in 16 out 27 cases (59.3%) a discordant result was reported (*n* = 11 MSS for Titano MSI kit and MSI-H for TapeStation 4200 and *n* = 5 MSI-H for Titano MSI kit and MSS for TapeStation 4200). The results are summarized in Table 6.

## 4. Discussion

The crucial role of MMR/MSI analysis for ICI administration followed the FDA approval in 2017 of the anti-PD-1 immune checkpoint monoclonal antibody pembrolizumab for MSI-H/dMMR patients with unresectable or metastatic solid tumors, regardless of age and histotype [29,30]. However, beyond the clear role in the management of CRC patients, the efficacy of ICIs in other solid tumors featuring MSI-H/dMMR status, including ECs, OCs, PaCs, PrCs, and GCs, has been demonstrated [31,32,33,34,35,36,37,38,39,40,41,42]. Thus, it is pivotal that MSI-H/dMMR status in different types of solid tumors be evaluated.

Although IHC remains the gold standard approach for MSI biomarker analysis in solid tumors, our data indicate that both Idylla and TapeStation molecular platforms are able to analyze MSI status just as efficiently as IHC. Indeed, a high concordance rate was observed between the Idylla™ platform and IHC (94.5% and 77.3% for the for MSS/pMMR and MSI-H/dMMR cases, respectively), and between the TapeStation 4200 microfluidic system and IHC (91.4% and 68.2% for the MSS/pMMR and MSI-H/dMMR cases, respectively). Of note, overall high concordance rates were also seen between the TapeStation 4200 and Idylla™ platforms (93.1% and 69.2% for MSS and MSI-H cases, respectively). In addition, in line with previous data, GCs (28.9%) and ECs (27.0%) showed the highest rates of dMMR cases, followed by PrCs (7.5%) and OCs (5.3%). In our experience, all PaC cases showed a pMMR profile. Similarly, MSI-H status was more frequent in GCs (28.2% and 28.2%, by Idylla^TM^ and TapeStation 4200, respectively) and ECs (33.3% and 24.3%, by Idylla^TM^ and TapeStation 4200, respectively) than in OCs (7.3% and 13.2%, by Idylla^TM^ and TapeStation 4200, respectively), PrCs (2.5% and 7.5%, by Idylla^TM^ and TapeStation 4200, respectively), and PaCs (0.0% and 5.7%, by Idylla^TM^ and TapeStation 4200, respectively).

Hence, our data clearly highlight that molecular analysis represents a valid upfront approach to evaluate MSI status in various types of cancer patients who would definitely benefit from ICI treatments. Indeed, we have demonstrated that MMR/MSI testing has acquired a central role not only in CRC management but also in the clinical stratification of different solid tumors [29,30]. In fact, patients displaying MSI-H/dMMR status respond well to immunotherapy regimens [29,30]. It is against this background that molecular analysis could represent a valuable strategy to overcome the well-known limitations of IHC, including intra- and inter-observer variability and pre-analytical and analytical issues [1]. In particular, a recent study has highlighted that a pMMR IHC profile may be retained in cases featuring antigenically intact non-functioning proteins despite the presence of an MSI-H status [44]. In addition, the high global concordance (91.4% and 68.2% for MSS/pMMR and MSI-H/dMMR cases, respectively) observed between the fully automated PCR high-resolution melt curve analysis (Idylla^TM^) and the automated microfluidic electrophoretic run chip-based assay highlights that these user-friendly, rapid, and sensitive molecular techniques are interchangeable and, therefore, all equally efficient. Moreover, in all cases they seemed to outperform the gold standard IHC approach in selecting MSI-H/dMMR advanced patients for ICI treatments. Remarkably, for each solid tumor analyzed, they were both able to identify a higher percentage of patients eligible for ICI administration than did IHC. Although these results clearly indicate the practical advantages of integrating these approaches into clinical practice, further studies are definitely warranted to confirm whether their implementation in clinical practice might actually help oncologists to choose the best treatment option for their patients.

Among the 27 discordant cases, 13 tumors showed different IHC and Titano profiling. Twelve of these tumors were endometrial and prostate adenocarcinomas. Of note, different molecular mechanisms characterize MSI in the different organs and may impact microsatellite instability testing and MMR IHC [45]. Focusing on EC, high agreement between IHC and MSI analysis has been described. However, several factors can affect this view: (i) MSH6 negative tumors may show MSS or MSI-L at MSI analysis [46]; (ii) *POLE* mutated tumors may present an MSI profile due to the accumulation of mutations in microsatellite loci despite preserved MMR protein expression; (iii) subclonal loss of MMR protein expression is present in around 3% of EC cases; a minimal microsatellite shift characterizes MSI EC and may interfere with the diagnostic interpretation of the results [47]; (iv) the use of obsolete IHC evaluation criteria may impact MMR evaluation [48]; and (v) the different PCR-based approaches are affected by intrinsic limitations that adequate training of the user and a high quality sample can overcome [15]. Overall, our data pinpointed a central role for molecular techniques in the diagnostic evaluation of MSI-H/dMMR status not only in CRC patients but also in other types of solid tumors. Future efforts should be focused on designing and developing clinical trials to validate the actual ability of molecular techniques to stratify patients with different types of solid tumors, including breast cancer [49,50], who may benefit from ICIs.

## Figures and Tables

**Table 1:** Summary of molecular analysis of MMR/MSS status for endometrial cancer patients.

ID	% Neoplastic Cells (I Evaluation)	% Neoplastic Cells (II Evaluation)	DNA Amount (ng/µL)	DIN	IHC	Idylla™	TapeStation 4200
1	90.00	80.00	205.00	3.50	pMMR	MSI-H	MSI-H
2	40.00	60.00	15.30	3.30	pMMR	MSS	MSS
3	70.00	70.00	7.99	2.20	pMMR	MSS	MSS
4	90.00	70.00	77.70	4.30	pMMR	MSS	MSS
5	25.00	30.00	172.00	5.60	pMMR	MSS	MSS
6	80.00	60.00	9.91	2.60	dMMR	MSS	MSI-H
7	70.00	60.00	133.00	2.90	pMMR	MSS	MSS
8	90.00	70.00	101.00	2.80	pMMR	MSS	MSS
9	80.00	70.00	6.58	1.90	pMMR	MSI-H	MSS
10	80.00	60.00	284.00	3.90	pMMR	MSI-H	MSI-H
11	80.00	70.00	14.70	2.10	pMMR	MSS	MSS
12	90.00	30.00	63.80	4.00	pMMR	MSI-H	MSI-H
13	70.00	70.00	67.80	4.30	pMMR	MSS	MSS
14	70.00	60.00	343.00	4.50	pMMR	MSS	MSS
15	80.00	60.00	19.50	3.30	pMMR	MSS	MSS
16	60.00	80.00	163.00	4.00	pMMR	MSS	MSS
17	80.00	60.00	31.60	4.20	pMMR	MSS	MSS
18	80.00	80.00	78.50	3.30	pMMR	MSS	MSS
19	70.00	60.00	253.00	4.90	pMMR	MSS	MSS
20	90.00	70.00	131.00	3.50	dMMR	MSI-H	MSI-H
21	80.00	70.00	119.00	2.10	pMMR	MSS	MSS
22	90.00	70.00	106.00	4.30	dMMR	MSI-H	MSS
23	50.00	60.00	103.00	3.80	pMMR	MSS	MSS
24	90.00	70.00	14.50	4.30	pMMR	MSI-H	MSI-H
25	90.00	60.00	29.40	3.30	pMMR	MSS	MSS
26	90.00	70.00	15.80	2.70	dMMR	MSI-H	MSS *
27	90.00	60.00	156.00	4.50	pMMR	MSI-H	MSI-H
28	70.00	60.00	78.90	2.80	dMMR	MSS	MSS
29	80.00	70.00	121.00	3.40	pMMR	MSS	MSS
30	90.00	50.00	45.10	3.20	pMMR	MSS	MSS
31	70.00	50.00	7.58	2.70	pMMR	MSS	MSS
32	80.00	80.00	16.80	3.20	pMMR	MSI-H	MSS *
33	60.00	60.00	6.84	2.40	dMMR	MSI-H	MSI-H

Note: * MSI-L. Abbreviations: DIN: DNA integrity number; dMMR: deficient mismatch repair; ID: identification number; IHC: immunohistochemistry; MSI-H: high microsatellite instability; MSI-L: low microsatellite instability MSS: microsatellite stable; pMMR: proficient mismatch repair.

**Table 2:** Summary of molecular analysis of MMR/MSS status for ovarian cancer patients.

ID	% Neoplastic Cells (I Evaluation)	% Neoplastic Cells (II Evaluation)	DNA Amount (ng/µL)	DIN	IHC	Idylla™	TapeStation 4200
1	35.00	20.00	117.00	5.90	pMMR	MSS	MSS
2	70.00	70.00	414.00	5.10	pMMR	MSS	MSS
3	80.00	40.00	89.40	5.80	pMMR	MSS	MSS *
4	80.00	60.00	118.90	5.30	pMMR	MSS	MSS *
5	80.00	70.00	374.00	5.10	pMMR	MSS	MSS
6	80.00	70.00	163.00	5.80	pMMR	MSS	MSS
7	40.00	30.00	48.00	4.60	pMMR	MSS	MSS *
8	90.00	60.00	254.00	5.30	pMMR	MSS	MSS
9	80.00	80.00	199.00	5.80	pMMR	MSS	MSI-H
10	90.00	70.00	83.50	4.70	pMMR	MSS	MSI-H
11	90.00	40.00	46.10	3.90	dMMR	MSI-H	MSI-H
12	90.00	60.00	25.40	3.80	pMMR	MSS	MSS
13	90.00	60.00	221.00	4.20	pMMR	MSS	MSS
14	90.00	80.00	191.00	3.90	pMMR	MSS	MSS *
15	70.00	50.00	48.30	2.40	pMMR	MSI-H	MSS *
16	90.00	60.00	147.00	4.60	pMMR	MSS	MSS
17	80.00	70.00	230.00	4.40	pMMR	MSS	MSS
18	90.00	60.00	112.00	4.50	pMMR	MSS	MSS
19	50.00	40.00	18.70	4.20	pMMR	MSS	MSS
20	90.00	80.00	93.80	3.70	pMMR	MSS	MSS
21	80.00	40.00	25.10	2.60	pMMR	MSS	MSS *
22	90.00	60.00	48.30	3.90	pMMR	MSS	MSS *
23	70.00	50.00	324.00	5.10	pMMR	MSS	MSS
24	90.00	70.00	424.00	4.30	pMMR	MSS	MSI-H
25	70.00	60.00	130.00	5.20	pMMR	MSS	MSS
26	80.00	60.00	79.40	5.20	pMMR	MSS	MSS
27	60.00	20.00	24.50	5.30	pMMR	MSS	MSS
28	80.00	60.00	41.80	5.70	pMMR	MSS	MSS
29	50.00	20.00	13.20	2.90	pMMR	MSS	MSS
30	90.00	70.00	30.80	3.60	dMMR	MSI-H	MSS
31	90.00	70.00	322.00	5.70	pMMR	MSS	MSS *
32	80.00	50.00	126.00	3.80	pMMR	MSS	MSS
33	60.00	60.00	218.00	5.10	pMMR	MSS	MSS
34	80.00	70.00	235.00	3.60	pMMR	MSS	MSS
35	90.00	70.00	45.90	2.20	pMMR	MSS	MSS
36	80.00	70.00	94.40	3.70	pMMR	MSS	MSS *
37	80.00	50.00	21.80	1.90	pMMR	MSS	MSS
38	90.00	80.00	13.40	2.00	pMMR	MSS	MSI-H

Note: * MSI-L. Abbreviations: DIN: DNA integrity number; dMMR: deficient mismatch repair; ID: identification number; IHC: immunohistochemistry; MSI-H: high microsatellite instability; MSI-L: low microsatellite instability MSS: microsatellite stable; pMMR: proficient mismatch repair.

**Table 3:** Summary of molecular analysis of MMR/MSS status for pancreas cancer patients.

ID	% Neoplastic Cells (I Evaluation)	% Neoplastic Cells (II Evaluation)	DNA Amount (ng/µL)	DIN	IHC	Idylla™	TapeStation 4200
1	80.00	30.00	11.60	2.50	pMMR	MSS	MSS
2	70.00	20.00	6.10	2.10	pMMR	MSS	MSS
3	85.00	50.00	3.00	2.40	pMMR	MSS	MSS
4	95.00	70.00	10.00	2.70	pMMR	MSS	MSS
5	90.00	30.00	3.30	2.30	pMMR	MSS	MSS
6	80.00	50.00	4.80	2.20	pMMR	MSS	MSS
7	90.00	70.00	5.80	2.00	pMMR	MSS	MSS *
8	95.00	40.00	14.60	2.00	pMMR	MSS	MSS
9	80.00	50.00	3.10	2.00	pMMR	MSS	MSS
10	95.00	60.00	8.10	2.00	pMMR	MSS	MSS
11	80.00	30.00	19.00	2.10	pMMR	MSS	MSS
12	90.00	70.00	114.00	2.40	pMMR	MSS	MSS
13	95.00	60.00	12.60	3.70	pMMR	MSS	MSS
14	95.00	50.00	56.70	3.00	pMMR	MSS	MSS
15	60.00	30.00	4.90	4.00	pMMR	MSS	MSS *
16	20.00	20.00	60.30	2.70	pMMR	MSS	MSS
17	90.00	80.00	146.00	3.30	pMMR	MSS	MSI-H
18	60.00	30.00	15.20	1.70	pMMR	MSS	MSS
19	70.00	60.00	4.50	1.80	pMMR	MSS	MSS
20	80.00	60.00	22.80	2.10	pMMR	MSS	MSS
21	90.00	70.00	2.40	0.00	pMMR	MSS	MSS *
22	20.00	40.00	3.20	1.20	pMMR	MSS	MSS
23	40.00	20.00	4.00	2.70	pMMR	MSS	MSS
24	90.00	70.00	182.00	4.80	pMMR	MSS	MSI-H
25	25.00	30.00	12.70	3.10	pMMR	MSS	MSS *
26	80.00	30.00	8.40	3.30	pMMR	MSS	MSS
27	80.00	50.00	28.60	3.50	pMMR	MSS	MSS
28	90.00	70.00	42.40	3.20	pMMR	MSS	MSS
29	90.00	80.00	18.40	3.20	pMMR	MSS	MSS
30	20.00	10.00	43.50	4.70	pMMR	MSS	MSS
31	80.00	50.00	4.50	3.00	pMMR	MSS	MSS
32	80.00	20.00	14.80	1.90	pMMR	MSS	MSS *
33	70.00	40.00	131.00	3.60	pMMR	MSS	MSS
34	90.00	70.00	27.70	4.80	pMMR	MSS	MSS
35	80.00	40.00	36.10	2.80	pMMR	MSS	MSS

Note: * MSI-L. Abbreviations: DIN: DNA integrity number; dMMR: deficient mismatch repair; ID: identification number; IHC: immunohistochemistry; MSI-H: high microsatellite instability; MSI-L: low microsatellite instability MSS: microsatellite stable; pMMR: proficient mismatch repair.

**Table 4:** Summary of molecular analysis of MMR/MSS status for prostate cancer patients.

ID	% Neoplastic Cells (I Evaluation)	% Neoplastic Cells (II Evaluation)	DNA Amount (ng/µL)	DIN	IHC	Idylla™	TapeStation 4200
1	80.00	50.00	12.20	4.00	pMMR	MSS	MSS
2	85.00	70.00	18.90	4.10	pMMR	MSS	MSS *
3	95.00	60.00	34.30	4.00	pMMR	MSS	MSS *
4	95.00	60.00	21.40	3.10	pMMR	MSS	MSS *
5	70.00	50.00	15.10	4.00	dMMR	MSS	MSI-H
6	85.00	40.00	4.09	3.00	pMMR	MSS	MSS *
7	85.00	40.00	16.30	3.90	pMMR	MSS	MSS
8	95.00	70.00	27.50	4.00	pMMR	MSS	MSS
9	95.00	70.00	56.10	4.20	pMMR	MSS	MSS
10	85.00	30.00	25.20	4.30	pMMR	MSS	MSS
11	85.00	70.00	14.10	3.50	pMMR	MSS	MSI-H
12	90.00	80.00	52.70	4.40	pMMR	MSI-H	MSS *
13	90.00	60.00	23.60	3.80	pMMR	MSS	MSS
14	80.00	60.00	13.80	4.10	pMMR	MSS	MSS *
15	90.00	70.00	42.10	3.10	pMMR	MSS	MSS
16	85.00	60.00	13.30	3.20	pMMR	MSS	MSS
17	90.00	80.00	23.10	3.70	pMMR	MSS	MSS
18	85.00	50.00	7.07	3.80	pMMR	MSS	MSS
19	90.00	70.00	58.30	3.90	pMMR	MSS	MSS*
20	85.00	70.00	25.90	3.40	pMMR	MSS	MSS
21	85.00	50.00	51.00	5.00	dMMR	MSS	MSS
22	80.00	60.00	38.20	4.70	pMMR	MSS	MSS
23	90.00	80.00	31.80	3.80	pMMR	MSS	MSS
24	90.00	80.00	52.40	3.70	pMMR	MSS	MSS
25	85.00	60.00	92.20	4.70	dMMR	MSS	MSS
26	85.00	80.00	30.80	3.70	pMMR	MSS	MSS
27	95.00	80.00	104.00	3.80	pMMR	MSS	MSS *
28	90.00	60.00	22.40	4.00	pMMR	MSS	MSS
29	90.00	70.00	13.40	2.80	pMMR	MSS	MSS *
30	90.00	60.00	10.90	2.70	pMMR	MSS	MSS
31	95.00	70.00	37.70	4.00	pMMR	MSS	MSS
32	90.00	60.00	38.50	3.90	pMMR	MSS	MSS
33	90.00	50.00	14.20	3.00	pMMR	MSS	MSS
34	90.00	50.00	18.90	2.60	pMMR	MSS	MSS *
35	95.00	70.00	23.30	4.60	pMMR	MSS	MSS *
36	80.00	50.00	9.67	3.40	pMMR	MSS	MSS *
37	90.00	50.00	179.00	5.30	pMMR	MSS	MSS
38	85.00	50.00	2.04	2.30	pMMR	MSS	MSS
39	90.00	70.00	14.50	3.80	pMMR	MSS	MSI-H
40	85.00	60.00	14.50	3.40	pMMR	MSS	MSS *

Note: * MSI-L. Abbreviations: DIN: DNA integrity number; dMMR: deficient mismatch repair; ID: identification number; IHC: immunohistochemistry; MSI-H: high microsatellite instability; MSI-L: low microsatellite instability MSS: microsatellite stable; pMMR: proficient mismatch repair.

**Table 5:** Summary of molecular analysis of MMR/MSS status for gastric cancer patients.

ID	% Neoplastic Cells (I Evaluation)	% Neoplastic Cells (II Evaluation)	DNA Amount (ng/µL)	DIN	IHC	Idylla™	TapeStation 4200
1	80.00	20.00	17.20	2.90	pMMR	MSS	MSS *
2	90.00	70.00	60.00	3.70	pMMR	MSS	MSS
3	80.00	60.00	65.80	4.80	pMMR	MSS	MSS
4	80.00	40.00	4.40	0.00	pMMR	MSS	MSS
5	90.00	60.00	145.00	5.30	pMMR	MSS	MSS *
6	90.00	50.00	12.30	3.20	pMMR	MSS	MSS
7	90.00	70.00	66.30	2.80	pMMR	MSS	MSS
8	90.00	70.00	92.60	5.50	dMMR	MSI-H	MSI-H
9	70.00	20.00	34.40	2.70	pMMR	MSS	MSS
10	90.00	60.00	84.60	3.30	pMMR	MSS	MSS
11	85.00	70.00	33.30	3.20	dMMR	MSI-H	MSI-H
12	70.00	30.00	88.30	3.80	pMMR	MSS	MSS
13	80.00	70.00	92.20	2.70	dMMR	MSI-H	MSI-H
14	60.00	40.00	50.40	4.60	pMMR	MSS	MSS
15	90.00	50.00	27.90	2.50	dMMR	MSI-H	MSI-H
16	95.00	60.00	10.50	3.70	dMMR	MSI-H	MSS
17	90.00	20.00	4.80	2.80	pMMR	MSS	MSS *
18	60.00	20.00	25.20	3.60	pMMR	MSS	MSS
19	90.00	70.00	68.00	2.40	pMMR	MSS	MSS
20	80.00	20.00	51.80	3.60	pMMR	MSS	MSS
21	90.00	60.00	187.00	4.20	dMMR	MSI-H	MSI-H
22	80.00	40.00	16.30	3.90	pMMR	MSS	MSS
23	90.00	70.00	109.00	3.00	pMMR	MSS	MSS
24	90.00	60.00	22.10	3.20	pMMR	MSS	MSS
25	80.00	30.00	45.70	3.60	pMMR	MSS	MSS *
26	85.00	60.00	10.30	3.80	dMMR	MSI-H	MSI-H
27	95.00	60.00	120.60	3.80	dMMR	MSI-H	MSI-H
28	90.00	50.00	111.00	4.20	pMMR	MSS	MSS
29	80.00	60.00	25.40	4.30	pMMR	MSS	MSS
30	85.00	70.00	41.30	3.10	pMMR	MSS	MSS *
31	85.00	70.00	57.30	3.50	dMMR	MSI-H	MSI-H
32	80.00	40.00	120.00	5.30	pMMR	MSS	MSS
33	95.00	70.00	64.40	4.40	dMMR	MSI-H	MSI-H
34	90.00	60.00	56.80	4.70	dMMR	MSI-H	MSI-H
35	95.00	80.00	85.90	2.50	pMMR	MSS	MSI-H
36	70.00	30.00	159.00	3.50	pMMR	MSS	MSS
37	90.00	50.00	93.20	4.40	pMMR	MSS	MSS
38	80.00	50.00	94.80	5.20	pMMR	MSS	MSS *
39	65.00	30.00	60.40	3.90	pMMR	MSS	MSS

Note: * MSI-L. Abbreviations: DIN: DNA integrity number; dMMR: deficient mismatch repair; ID: identification number; IHC: immunohistochemistry; MSI-H: high microsatellite instability; MSI-L: low microsatellite instability MSS: microsatellite stable; pMMR: proficient mismatch repair.

**Table 6:** Comparative analysis of the discordant cases.

Case ID	Titano	IHC	Idylla^TM^	TapeStation 4200
EC 1	MSI-H	pMMR	MSI-H	MSI-H
EC 6	MSI-H	dMMR	MSS	MSI-H
EC 11	MSS *	pMMR	MSI-H	MSS
EC 12	MSS *	pMMR	MSI-H	MSI-H
EC 14	MSI-H	pMMR	MSI-H	MSI-H
EC 28	MSI-H	dMMR	MSI-H	MSS
EC 30	MSI-H	pMMR	MSI-H	MSI-H
EC 32	MSI-H	dMMR	MSI-H	MSS *
EC 33	MSI-H	pMMR	MSI-H	MSI-H
EC 34	MSS*	dMMR	MSS	MSS
EC 38	MSI-H	pMMR	MSI-H	MSS *
OC 9	MSS	pMMR	MSS	MSI-H
OC 10	MSS	pMMR	MSS	MSI-H
OC 15	MSS *	pMMR	MSI-H	MSS *
OC 24	MSS	pMMR	MSS	MSI-H
OC 31	MSS	dMMR	MSI-H	MSS
OC 39	MSS	pMMR	MSS	MSI-H
PaC 19	MSS	pMMR	MSS	MSI-H
PaC 27	MSS	pMMR	MSS	MSI-H
PrC 5	MSS	dMMR	MSS	MSI-H
PrC 11	MSS	pMMR	MSS	MSI-H
PrC 12	MSI-H	pMMR	MSI-H	MSS *
PrC 21	MSS	dMMR	MSS	MSS
PrC 25	MSS	dMMR	MSS	MSS
PrC 39	MSS	pMMR	MSS	MSI-H
GC 16	MSI-H	dMMR	MSI-H	MSS
GC 36	MSS	pMMR	MSS	MSI-H

Note: * MSI-L. Abbreviations: dMMR: deficient mismatch repair; EC: endometrial carcinoma; GC: gastric carcinoma; ID: identification number; IHC: immunohistochemistry; MSI-H: high microsatellite instability; MSI-L: low microsatellite instability; MSS: microsatellite stable; OC: ovarian carcinoma; PaC: pancreatic carcinoma; PrC: prostatic carcinoma; pMMR: proficient mismatch repair.

## Data Availability

The data presented in this study are available on request from the corresponding author.
